# A new species of Tequila Giant Skipper *Aegiale* C. Felder & R. Felder, 1860 (Lepidoptera, Hesperiidae, Megathymina) from Sonora, Mexico

**DOI:** 10.3897/zookeys.1272.181963

**Published:** 2026-03-05

**Authors:** Sajan KC, William W. McGuire, Keith R. Willmott, Anisha Sapkota, Vaughn M. Shirey

**Affiliations:** 1 McGuire Center for Lepidoptera and Biodiversity, Florida Museum of Natural History, University of Florida, Gainesville, FL 32611, USA University of Florida Gainesville United States of America

**Keywords:** Agave, desert fauna, Grass Skippers, monotypic, Neotropical butterflies

## Abstract

*Aegiale* C. Felder & R. Felder, 1860 has traditionally been treated as a monotypic genus of Giant Skippers (Hesperiidae: Megathymina), represented by *A. hesperiaris* (Walker, 1856), which occurs from northern to central Mexico, mostly east of the Sierra Madre Occidental mountain range. Here, we describe a second species, *Aegiale occidentalis* KC, McGuire & Shirey, **sp. nov**., from Sonora, Mexico, occurring in the Sierra Madre Occidental. Morphological comparisons, including female genitalia, were used to diagnose the new species. *Aegiale occidentalis***sp. nov**. differs from *A. hesperiaris* in dorsal forewing coloration, with fully orange apical and discal cell spots, and in female genitalia, including shorter papillae anales and a reduced sterigma. We provide illustrations of the habitus and female genitalia of both species, a key to differentiate them, and a key to distinguish *Aegiale* from other Megathymina genera. The discovery of *A. occidentalis***sp. nov**. expands the known diversity of the genus and highlights underexplored montane regions in northwestern Mexico, with implications for the biogeography of agave-feeding Megathymina.

## Abstract (de)

*Aegiale* C. Felder & R. Felder, 1860 ha sido tradicionalmente tratado como un género monotípico de saltadores gigantes (Hesperiidae: Megathymina), representado por *A. hesperiaris* (Walker, 1856), que se distribuye desde el norte hasta el centro de México, principalmente al este de la Sierra Madre Occidental. Aquí describimos una segunda especie, *Aegiale occidentalis* KC, McGuire & Shirey, **sp. nov**., de Sonora, México, que se encuentra en la Sierra Madre Occidental. Se utilizaron comparaciones morfológicas, incluyendo la genitalia femenina, para diagnosticar la nueva especie. *Aegiale occidentalis***sp. nov**. se diferencia de *A. hesperiaris* en la coloración dorsal del ala anterior, con manchas apicales y de la celda discal completamente naranjas, y en la genitalia femenina, incluyendo papilas anales más cortas y un esterigma reducido. Proporcionamos ilustraciones del hábitus y de la genitalia femenina de ambas especies, una clave para diferenciarlas y una clave para distinguir *Aegiale* de otros géneros de Megathymina. El descubrimiento de *A. occidentalis***sp. nov**. amplía la diversidad conocida del género y destaca regiones montañosas poco exploradas en el noroeste de México, con implicaciones para la biogeografía de los Megathymina que se alimentan de agave.

## Introduction

The Giant Skippers (Lepidoptera, Hesperiidae, Megathymina) are a distinctive group of skipper butterflies primarily found in the arid regions of North America (Barnes and McDunnough 1912; Evans 1955; Roever 1964; Freeman 1969). To date, as many as 39 species and approximately 24 additional subspecies have been recognized (Freeman 1967, 1969; Roever 1975; Scott 1986; Warren et al. 2024). Broadly, the group is divided into two ecological lineages: the *Megathymus* group, which includes the genera *Megathymus* Scudder, 1872 and *Stallingsia* Freeman, 1959, whose larvae feed mostly within yucca or tuberose roots (Asparagaceae) and construct silken tents as exit structures; and the *Aegiale* group, which includes the genera *Aegiale* C. Felder & R. Felder, 1860, *Turnerina* Freeman, 1959, and *Agathymus* Freeman, 1959, whose larvae feed mostly within agave leaves (Asparagaceae) and construct trapdoors as exit structures (Freeman 1969; Scott 1986). Within the agave-feeding lineage, the genus *Aegiale* has traditionally been considered monotypic, comprising only *Aegiale hesperiaris* (Walker, 1856) (Evans 1955; Freeman 1959, 1969; KC and Sapkota 2024; Warren et al. 2024). Known as the Tequila Giant Skipper, larvae of *A. hesperiaris* feed internally on the leaves of various species of *Agave* (Asparagaceae) (Jaimes-Rodríguez et al. 2020; Molina-Vega et al. 2021; KC and Sapkota 2024). The larvae, also known as ‘gusano blanco de maguey’ and ~11 other local names (see Ramos-Elorduy et al. 2011), are harvested from inside the agave leaves and consumed as a delicacy in various parts of Mexico, either raw, cooked, or incorporated into alcoholic beverages such as mezcal (Ramos-Elorduy 2006; Molina-Vega et al. 2021; Piña-Domínguez et al. 2022; Herrera-Cardoso et al. 2025).

Currently, *A. hesperiaris* is known only from Mexico, with confirmed records primarily from the northern and central regions well to the east of the Sierra Madre Occidental. However, since its larval host plants are far more widely distributed across similar habitats than the currently known range of the skipper (iNaturalist 2025), it has been speculated that the species may occur elsewhere, with unconfirmed reports even from Texas, USA, possibly resulting from occasional introductions (Bordelon and Knudson 2006; KC and Sapkota 2024).

In this paper, we describe a new species of *Aegiale* from Sonora State, Mexico – collected from the Sierra Madre Occidental – based on a single female specimen. Diagnostic characters distinguishing the new species from *A. hesperiaris* are presented, based both on wing and female genitalia morphology. A species distribution model for *Aegiale* is also provided using distributional data of *A. hesperiaris* obtained from the Global Biodiversity Information Facility (GBIF) and the examined specimen labels to infer potential habitat suitability within the region.

## Materials and methods

The following abbreviations are used for institutions mentioned in this paper:

**MGCL** McGuire Center for Lepidoptera and Biodiversity, Florida Museum of Natural History, University of Florida, Gainesville, Florida, USA;

**FSCA** Florida State Collection of Arthropods, Division of Plant Industry, Gainesville, Florida, USA;

**NHMUK** The Natural History Museum, London, UK.

**Figure 1.**
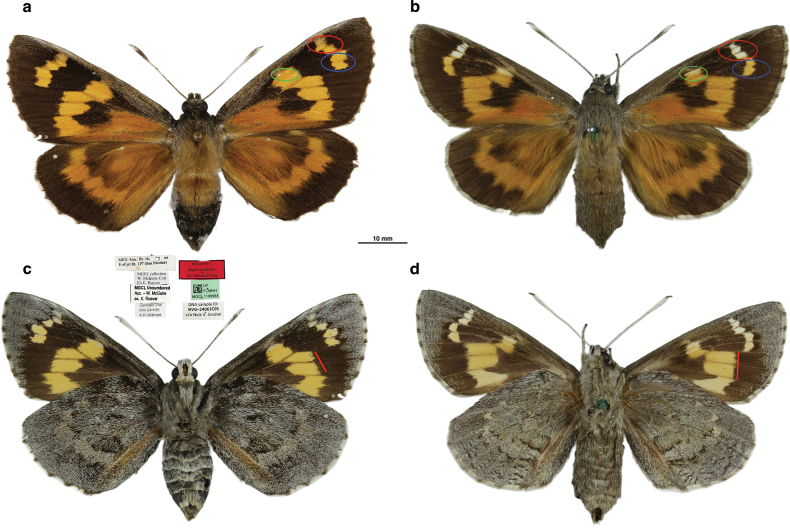
Comparative dorsal (**a, b**) and ventral (**c, d**) views of *Aegiale* females, illustrating key morphological differences. **a, c**. Holotype of *A. occidentalis* sp. nov.; **b, d**. *A. hesperiaris* (specimen no. 37680).

**Figure 2.**
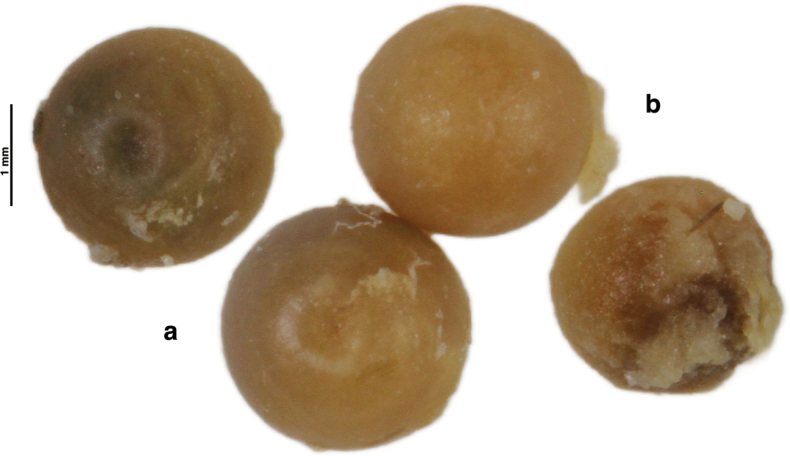
Eggs extracted from the holotype of *Aegiale occidentalis* sp. nov. during genitalia examination. **a**. Dorsal; **b**. Ventral.

**Figure 3.**
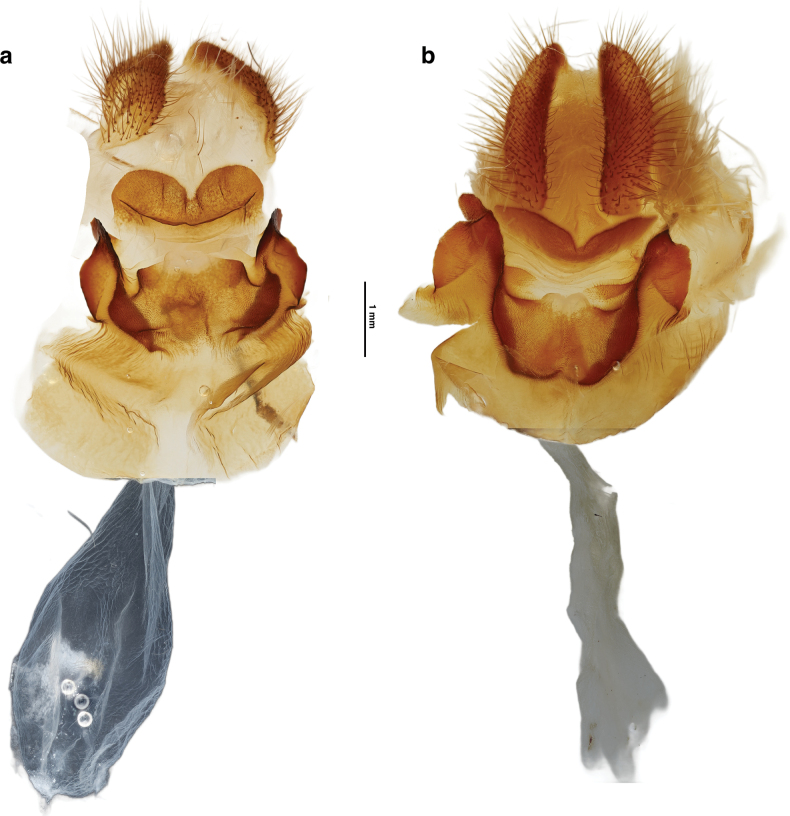
Comparative female genitalia morphology of *Aegiale* species in ventral views. **a**. Holotype of *A. occidentalis* sp. nov.; **b**. *A. hesperiaris* (specimen no. 37679).

All of the specimens physically examined in this study are deposited in MGCL. The single specimen of *A. occidentalis* sp. nov. was discovered in a series of specimens of Kilian Roever’s former collection during curatorial work by the fourth author (AS). The specimens (Figs 1, 2) were photographed using a Canon EOS 7D digital camera equipped with a Canon EF-S 60 mm f/2.8 Macro USM lens. Genitalic examinations were conducted by soaking the abdomens overnight in 10% potassium hydroxide (KOH) at room temperature. Leg morphology was studied following a 1-min soak in concentrated bleach. All morphological structures were examined under a Leica MZ16 stereomicroscope. A Macropod imaging system equipped with a Canon EOS 6D Mark II camera and a Canon MP-E 65 mm macro lens at FSCA was used to capture genitalia images (Fig. 3) of the specimens. Focus stacking was performed using Canon EOS Utility v3.14.30.4 and Helicon Focus® Pro v7.7.5. Additional post-processing was conducted using Photopea (https://www.photopea.com/) and the Preview application on macOS (v11.0).

Attempts were made to sequence the COI ‘barcode’ region of the holotype. Genomic DNA was extracted from the abdomen using the Qiagen DNEasy Blood and Tissue Kit, following the manufacturer’s protocol except quadrupling the volume of reagents to accommodate the size of the abdomen. PCR was performed using the primers LCO/HCO (Folmer et al. 1994) using the methods followed by Willmott et al. (2017). Subsequent nested PCRs were also conducted using primer pairs LCO/K699 (Monteiro and Pierce 2001) and Ron/Nancy (Simon et al. 1994). Additional efforts were made to barcode DNA from eggs found within the abdomen using LCO and HCO primers. However, none of these attempts yielded successful amplification. A hindleg was sent to Nick V. Grishin at the Grishin Lab, University of Texas Southwestern Medical Center, for a further attempt to sequence the DNA; if successful, the resulting sequence may be published in the future.

**Figure 4.**
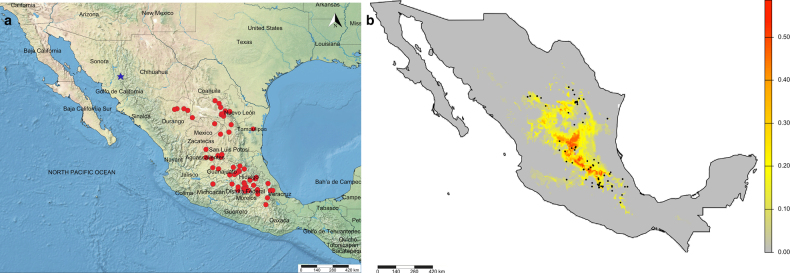
Distribution maps of *Aegiale*. **a**. Geographic distribution of *Aegiale occidentalis* sp. nov. (blue star) and *A. hesperiaris* (red circles) based on GBIF occurrence records and label data from examined specimens; **b**. Species distribution model for *A. hesperiaris* based on GBIF occurrence data, specimen label data, and a Bioclim model, with black dots (corresponding to red circles in Fig. 4a) indicating recorded localities. In the heatmap, gray indicates the least suitable habitat and red indicates the most suitable.

A distribution map (Fig. 4a) was generated using occurrence data from GBIF and georeferenced specimen label data via Google Maps, with the coordinates exported and visualized using SimpleMappr (https://www.simplemappr.net/). To generate the species distribution model (Fig. 4b), occurrence records of *A. hesperiaris* were compiled from examined specimen labels georeferenced using Google Maps, as well as from GBIF records accessed via the ‘rgbif’ package (Chamberlain et al. 2022) in RStudio v2024.12.1+563 (Posit Team 2024). Records on GBIF without coordinate data were excluded. Nineteen bioclimatic variables were downloaded from WorldClim v2.1 at 5 arc-minute resolution using the ‘geodata’ package and clipped to the extent of Mexico using shapefiles from Natural Earth (https://www.naturalearthdata.com). After excluding records with missing environmental data, resulting from invalid or incomplete coordinates, a Bioclim model was constructed using the ‘dismo’ package (Hijmans et al. 2017). The model was projected across the masked bioclimatic raster to generate a continuous habitat suitability map. Predictions were visualized using a custom heatmap color gradient in R, with warm colors representing higher predicted suitability. Model evaluation was performed using the evaluate function in the ‘dismo’ package, which compared predicted suitability at presence points and 10,000 randomly sampled background points. The model achieved an AUC (Area Under the Curve) of 0.923, indicating a good fit.

Label data of type specimens are provided verbatim, with line breaks indicated by a single forward slash and space (/ ), and separate labels indicated by double forward slashes with a space before and after ( // ). Square brackets ([ ]) are used for additional comments or emphasis. All labels are printed in black on white paper unless otherwise noted in square brackets.

Length measurements were taken as follows: **head**: from the tip of the palpus to the anterior margin of the pronotum; **thorax**: from the anterior margin of the pronotum to the posterior margin of the metanotum; **abdomen**: from the anterior margin of segment I to the posterior margin of the last visible segment including the end of the scales covering this segment; **antennae**: from the tip of the apiculus to the base of antenna; **wings**: from the center of the thorax to the apices of wings (Evans 1932). Width measurements were taken at the widest point of each body part, with head width measured across both eyes. The nomenclature of wing venation is based on the Comstock-Needham system (Comstock 1918) and Scott (1986), with cells referred to by their anterior bounding vein, while that of the genitalia is based on Klots (1970).

## Results

### Family Hesperiidae Latreille, 1809

**Subfamily Hesperiinae Latreille, 1809**

**Tribe Megathymini J. Comstock & A. Comstock, 1895**

**Subtribe Megathymina J. Comstock & A. Comstock, 1895**

#### Genus Aegiale

Taxon classificationAnimaliaLepidopteraHesperiidae

C. Felder & R. Felder, 1860

F90E9554-A268-593C-9DE2-FF3A63B66C7A

##### Type species.

*Aegiale kollari* C. Felder & R. Felder, 1860.

#### Aegiale occidentalis

Taxon classificationAnimaliaLepidopteraHesperiidae

KC, McGuire & Shirey sp. nov.

B69B10F8-44E3-5924-846A-AE13B77E3667

https://zoobank.org/80FB045C-D332-48A5-82AF-E991F6FE7909

Figs 1 a, c, 2, 3a, 4 Suggested English name: Western Tequila Giant Skipper.

##### Material examined.

***Holotype*** • ♀, Mexico: ‘MEX: Son.: Rt. 16, [handwritten, black ink] 7 [/handwritten, black ink] mi/ E of jct Rt. 117 (San Nicolas) // [underside of first label; handwritten, black ink] 6/20/92 // MGCL collection/ W. McGuire Coll./ Ex K. Roever // MGCL Unnumbered/ Acc. – W. McGuire/ ex. K. Roever // Genitalic Vial/ KW-24-135/ K.R. Willmott // [red label with black border] HOLOTYPE ♀/ *Aegiale occidentalis*/ KC, McGuire & Shirey **//** [green barcode label] UF/ FLMNH/ MGCL 1199968 // DNA sample ID:/ NVG-24063C05/ c/o Nick V. Grishin’ in MGCL (Fig. 1a, c).

##### Diagnosis.

*Aegiale occidentalis* sp. nov. can be distinguished from *A. hesperiaris* based on several morphological characters. 1) On the dorsal forewing, *A. occidentalis* sp. nov. exhibits dark orange apical spots whose inner margins are not aligned, with the basal edge of spots M_1_ and M_2_ not curving inward. In contrast, *A. hesperiaris* displays three white to whitish anterior apical spots whose inner margins are aligned or nearly so, with spots M_1_ and M_2_ pale orange and with their inner margins characteristically curved inward. 2) Spots CuA_1_ and M_3_ are obliquely inclined toward the costa in *A. occidentalis* sp. nov., whereas they are more vertical in *A. hesperiaris*. 3) The forewing discal cell spot in *A. occidentalis* sp. nov. is entirely orange whereas, in *A. hesperiaris*, the upper portion is white to whitish. 4) Ventrally, *A. occidentalis* sp. nov. is a darker gray, while *A. hesperiaris* is paler gray. 5) In dorsal view, the papillae anales in female genitalia are ~2× longer than wide in *A. occidentalis* sp. nov., while they are > 3× longer than wide in *A. hesperiaris*. 6) The sterigma is overall shorter with the lower margin unsclerotized and the upper margin with two round humps in *A. occidentalis* sp. nov., while it is longer with all margins well sclerotized and the upper margin concave in *A. hesperiaris*.

##### Description.

**Female** (Holotype). **Dorsal** (Fig. 1a). ***Head***. Narrower than thorax; 5 mm wide and 4.8 mm long; antennae mostly scaled white, some dark scales around clubs, 19 mm long extending beyond middle of forewing costa, with long, slender clubs and short 6-segmented orange brown apiculi; eyes black; palpi short; scales salt-and-pepper dorsally. ***Thorax***. 12.5 mm long and 10 mm wide; pronotum with dark gray scales; mesonotum laterally lighter gray and medially covered with long ochraceous/orange scales; metanotum mostly covered with ochraceous scales. ***Wings***. Forewing 44 mm long; background blackish brown; base covered with long orange scales; orange area below discal cell extending from base to discal area and running along dorsum merging with discal band; discal orange band extending from space CuA_2_ to discal cell spot, uniformly orange but lighter than basal orange patch; lower portion of spot CuA_2_ longer than upper portion; spot CuA_2_ (shorter) and CuA_1_ (longer) offset forming an acute notch at inner margin; spots CuA_1_ and M_3_ (subequal) inwardly arrow-shaped, completely aligned; spot M_3_ connected to discal cell spot forming an obtuse notch at distal margin; discal cell spot single without prominent cleft; subapical spots M_2_–R_3_ unevenly conjoined; spots M_2_ and M_1_ (subequal) completely aligned; spot R_5_ (shorter) approximately half aligned with spots M_1_ and R_4_; spots R_4_ and R_3_ aligned by more than half; spots M_2_–R_4_ uniformly orange, spot R_3_ somewhat paler, suffused with costal scales; costa covered with gray overscales; cilia checkered black and white. Hindwing length 35 mm; background color same as forewing; base to postdiscal area covered with long orange scales which merge with postdiscal band but continue along dorsum to submargin; orange oval discal cell spot obscured by long orange overscales; space 1A+2A pale orange from base to near submargin; postdiscal orange band curved inward, extending from space CuA_2_ to space Sc+R_1_; spots of uneven length; spots M_2_ and M_1_ subequal and fully aligned; rest of discal spots shorter; spot Sc+R_1_ as a faint patch; spots M_1_ (long) and Rs (short) approximately half aligned with the band forming an obtuse notch outward; spots M_3_ (short) and M_2_ (long) approximately half aligned with the band forming a right-angle notch outward; cilia checkered black and white. ***Abdomen***. 16 mm long and 10 mm wide; segment I-II fully covered in orange scales; segment III medially covered in orange scales; segment IV orange patch sparse; remaining areas covered in black and brownish scales; end of segments lined with brownish scales which become more profuse after segment V.

**Ventral** (Fig. 1c). ***Head***. Palpi white mixed with sparse dark gray scales; proboscis brown, 20 mm long. ***Thorax***. Covered with long hair-like scales; prothorax with a dark gray band; rest of thorax with dark gray scales. ***Legs*** brown covered with similar gray scales; tuft of woolly hair-like scales on inner sides of femur and tibiae; femurs mostly brown; tibiae and tarsi with continuous series of strong brown spines on inner sides extending to a lateral side except for foretibia which only has an epiphysis; tarsi ending with bifid claws; pulvillus large, semicircular, pad-like; tarsal formula 5-5-5; tarsal spur formula 0-2-2; femur longest in midleg and shortest in hindleg; overall midleg longest, foreleg shortest. ***Wings***. Forewing with blackish brown background; gray overscales run across costal margin, more pronounced and widened at apical portion and narrowed along termen; spots same as on dorsal side; discal spots M_3_ and CuA_1_ narrowly separated from each other and remaining spots by dark veins; apical spots more widely separated. Hindwing almost fully clothed in dark gray overscales except some black patches; dorsal spots reflected as gray muted spots black-banded on inner margins; two prominent black patches along costa at basal and discal portion; a strong pale orange bar runs along vein 1A+2A to postdiscal region. ***Abdomen*** with mostly dark gray scales; smudges of black scales mostly along margins of V and VI segments; tip covered with gray scales; segment VIII concealed within and attached to sterigma.

**Eggs** (Fig. 2): Dome-shaped; 2.5 mm diameter, with ~0.6 mm circular depression on top; color amber in the museum specimen, potentially whitish in the field (the holotype was a gravid female whose eggs were observed during genitalia preparation).

**Female genitalia** (Fig. 3a): Papillae anales almond-shaped, ~2× longer than wide, with rows of long dense setae; sterigma sclerotized; fold below papillae anales (upper fold) with a broadly convex hump on either side separated by a median cleft; a sclerotized lower margin borders fold except at terminals; lower fold with a lateral lobe extending on each side that widens in middle; upper margin of inner fold with square corners, sclerotization diminishing toward center of margin; lower margin lined by broadly convex folds at sides connected to lateral folds; bottom of lower margin not lined by sclerotization; ostium ventrally opening; ductus and corpus bursae membranous with no visible signa.

**Male**. Unknown.

##### Distribution

(Fig. 4): Known only from the type locality in San Antonio, Sonora State, Mexico. The coordinates (28.415, -109.110) are estimated from specimen label data and are not GPS-derived. The elevation of the type locality is estimated to be ~1170 m.

##### Flight.

June, potentially also in August (see Bailowitz et al. 2017 and discussion below).

##### Etymology.

The species epithet *occidentalis* is treated as a noun in apposition, derived from Latin *occidentalis* meaning ‘western,’ referring to the species’ known distribution in the Sierra Madre Occidental of western Mexico.

##### Deposition.

The holotype is deposited in the MGCL collection.

##### Holotype condition.

The holotype is in good condition except that the abdomen was removed for genitalia analysis, the proboscis was removed for measurement, and the hindlegs were removed for DNA sequencing. The genitalia and abdomen are preserved in a separate collection in glycerin and labeled with a unique number (KW-24-135) corresponding to the specimen, while the proboscis and a bleached portion of the foreleg are pinned beneath the specimen in a capsule.

##### Remarks.

Based on the original descriptions and available images and illustrations of the type material for each name, we are confident that none of the species-level names currently associated with *Aegiale hesperiaris* apply to the species described here. In *Aegiale kollari* C. Felder & R. Felder, 1860 (original description: p. 111; table 2, fig. 3) and *Teria agavis* P. Blásquez & I. Blásquez, 1865 (original description: p. 21; Lam. 2a), the white apical and costal spots diagnostic of *A. hesperiaris* are either clearly illustrated in the original figures or stated in the descriptions. *Castnia hesperiaris* Walker, 1856 was described without an accompanying illustration; however, Walker explicitly noted the presence of “two irregular oblique pale testaceous subcostal streaks” (original description: p. 1583), consistent with the pale costal markings characteristic of *A. hesperiaris*. We are confident that the image of the type specimen available on Butterflies of America (Warren et al. 2024) corresponds to one of Walker’s syntypes. None of these names corresponds to *A. occidentalis* sp. nov., which differs in having the apical and costal spots entirely orange, in addition to other diagnostic wing-pattern differences outlined in the Diagnosis and illustrated in Fig. 1.

#### Aegiale hesperiaris

Taxon classificationAnimaliaLepidopteraHesperiidae

(Walker, 1856)

CFEE8FB6-1B64-50B2-B065-C77CC7F51832

Figs 1 b, d, 3b, 4 English name: Tequila Giant Skipper.

Castnia hesperiaris Walker, 1856: 1583; Aegiale kollari C. Felder & R. Felder, 1860: 111; Teria agavis P. Blásquez & I. Blásquez, 1865: 21; Acentrocneme kollari Scudder, 1875: 100; Aegiale hesperiaris Riley, 1876: 341; Acentrocneme hesperiaris Kirby, 1877: 829.

##### Type locality.

Mexico.

##### Type material.

***Syntype***, • sex unknown, Mexico: ‘[circular label with red margin] Type // [circular label; handwritten, black ink] Mexico // SYNEMON THERESA. // 42. CASTNIA HESPERIARIS. // [circular label; handwritten, black ink] H/ 2533 // BMNH(E) #810380’, in NHMUK; image examined from Warren et al. (2024).

##### Material examined.

• 5 specimens: **Mexico**. • 1 ♀; D.F. [now Mexico City]; ?.x.1961; T. Escalante leg.; KW-24-144; MGCL/FLMNH 37680 (Fig. 1b, d); • 1 ♀; D.F. [now Mexico City]; San Pedro Xalpa; ?.ix.1958; T. Escalante leg.; KW-24-121; MGCL/FLMNH 37679 (Fig. 3b); • 1 ♂; Hidalgo; Apam [sic; recte Apan]; ?.ix.1959; T. Escalante leg.; MGCL 1189089; • 1 ♂; Hidalgo; Ixmiquilpan; ?.x.1972; B. Hollenbach leg.; DNA voucher LEP-33740; MGCL 1189090; • 1 ♂; Unknown locality; KW-24-143; MGCL 1189091.

##### Diagnosis.

*Aegiale hesperiaris* differs from *A. occidentalis* sp. nov. in the characters listed above in the diagnosis of the latter species, from which it can be distinguished by having white to whitish dorsal forewing apical spots whose inner margins are aligned or nearly so, the basal edges of apical spots M_1_ and M_2_ curved inward, a forewing discal cell spot with a white to whitish upper portion, paler gray ventral wing surface, female papillae anales more than three times longer than wide, and a longer, fully sclerotized sterigma with a concave upper margin.

**Female genitalia** (Fig. 3b): Papillae anales elongate kidney-shape, more than three times longer than wide, with rows of long dense setae; sterigma sclerotized; upper fold below papillae anales broadly V-shaped or concave with a median cleft; W-shaped lower fold thickens at the margins, eye-like folds inside on each side without any sclerotization in the center; an ear-shaped fold extending laterally on each side; ostium ventrally opening; ductus and corpus bursae membranous with small patches of signa; the entire vaginal plate shaped like a goat’s head with sterigma as the face and papillae anales as the horns.

##### Distribution

(Fig. 4): Primarily occurs in the arid regions of northern and central Mexico, mostly well east of the Sierra Madre Occidental mountain range (Freeman 1969; Warren et al. 2024; iNaturalist 2025) but has also been reported from localities along and around the Sierra Madre Occidental (de la Maza 1987; Bailowitz et al. 2017; iNaturalist 2025).

##### Hostplants.

The larvae of *A. hesperiaris* feed internally on the leaves of various species of *Agave* (Asparagaceae), including *A. americana* L., *A. atrovirens* Karw. ex Salm-Dyck, *A. lehmannii* Jacobi, *A. mapisaga* Trel., *A. salmiana* Otto ex Salm-Dyck, and *A. tequilana* F.A.C. Weber var. blue (Jaimes-Rodríguez et al. 2020; Molina-Vega et al. 2021; KC and Sapkota 2024).

##### Flight.

Adults are known to fly from April to December in Mexico (Freeman 1969; de la Maza 1987; Jaimes-Rodríguez et al. 2020; iNaturalist 2025), although iNaturalist (2025) records suggest that the species may occur year-round.

### Dichotomous key to identify adult *Aegiale* with respect to other Giant Skippers based on external morphology

**Table:** 

1	Antennae without distinct apiculi	***Megathymus* Scudder, 1872, *Agathymus* Freeman, 1959**
–	Antennae with distinct apiculi	**2**
2	Ventral hindwing with a strong band	***Turnerina* Freeman, 1959**
–	Ventral hindwing without a strong band	**3**
3	Dorsal wings without bright orange patches; ventral wings without thick gray overscaling	***Stallingsia* Freeman, 1959**
–	Dorsal wings with bright orange patches; ventral wings with thick gray overscaling	**4 *Aegiale* C. Felder & R. Felder, 1860**
4	Dorsal forewing with fully orange spots; lower two subapical spots not curved inward (Fig. 1a); ventral hindwing darker gray (Fig. 1c)	***A. occidentalis* sp. nov**.
–	Dorsal forewing with top discal cell spot and top three apical spots white or whitish; lower two subapical spots curved inward (Fig. 1b); ventral hindwing paler gray (Fig. 1d)	***A. hesperiaris* (Walker, 1856)**

## Discussion

The placement of *Aegiale occidentalis* sp. nov. within the genus *Aegiale* is supported by a combination of morphological characters shared with *A. hesperiaris*, the only other described species in the genus. Both species exhibit the defining traits of *Aegiale*, including distinctly elongated apiculi on the antennae, bright dorsal orange wing patches, and a ventral hindwing clothed in thick gray overscaling – characters that readily separate the genus from other Megathymina genera. Additionally, the general structure of the female genitalia in *A. occidentalis* sp. nov., including the presence of a bilobed upper sterigma fold and a ventrally opening ostium, is consistent with that of *A. hesperiaris*. Although molecular data could not be obtained, the morphological continuity in these key diagnostic features strongly supports inclusion in *Aegiale*. To confirm the stability of these characters in *A. hesperiaris*, we examined and dissected two female specimens from Mexico City. These dissections revealed minimal variation in genitalic morphology, particularly in the shape of the papillae anales and configuration of the sterigma, reinforcing the distinctiveness of the new species and the diagnostic value of these traits within the genus.

Occurrence data for *A. hesperiaris* from GBIF, together with examined museum specimens, indicate that the species is primarily distributed in central Mexico, with fewer records from western regions. All verified records are largely concentrated in northern and central Mexico (Fig. 4a), despite the widespread availability of its host plants throughout Mexico and into the southern United States, including Arizona and Texas. This pattern suggests that the two *Aegiale* species could potentially be geographically isolated by the Sierra Madre Occidental, with *A. hesperiaris* occurring in other mountains in Mexico east of the Sierra Madre Occidental, while *A. occidentalis* sp. nov. appears to be confined along the Sierra Madre Occidental, potentially down to Jalisco in the Transmexican Volcanic Belt. Nevertheless, ‘*A. hesperiaris*’ has been reported from Sonora (Sierra Madre Occidental) by Bailowitz et al. (2017), from Yécora (approximately 14 km NE from the type locality of *A. occidentalis* sp. nov.) in 1990 and near Trinidad Mine (approximately 70 km N) in 1986. Additional evidence for occurrence in Sonora is provided by Brock (2015), who included the eastern portion of Sonora within the distribution range of *A. hesperiaris*. Likewise, there are records from Jalisco as well in the Transmexican Volcanic Belt (de la Maza 1987; Vargas et al. 1996; Molina-Vega et al. 2021). While all these records may represent misidentified individuals of *A. occidentalis* sp. nov., as no supporting images or voucher specimens could be located, it is also possible that *A. occidentalis* sp. nov. is sympatric with *A. hesperiaris* along parts of its range. Additionally, the species distribution model for *A. hesperiaris* extends weakly along the Sierra Madre Occidental (Fig. 4b), suggesting the potential presence of the species in this region. Future research, including targeted field surveys, is required to clarify the distributions of these two *Aegiale* species and to determine the extent of potential sympatry along their distribution ranges.

Currently, nothing is known about the natural history or biology of *A. occidentalis* sp. nov., but it is expected to be similar to that of *A. hesperiaris*, which is found at similar elevations. Additional specimens may exist in museum collections across Mexico and the United States; however, given the pristine condition of the holotype and the clear morphological differences observed, we consider the available material sufficient to justify describing this species. While male genitalia are often considered more reliable for hesperiid identification (Evans 1949, 1955), Freeman and Hoffmann (1952) and Freeman (1955) noted that female genitalia in *Megathymus* – and potentially across all Megathymina – may be more dependable for species delimitation owing to lower intraspecific variation; a similar view was expressed by Barnes and McDunnough (1912).

With the habitats of agave species under threat in Mexico, affecting various species of insects associated with them (Delgado-Lemus et al. 2014; Alducin-Martínez et al. 2022), formally recognizing this taxon is important for its potential future conservation. In addition, the larvae of the *A. hesperiaris* complex are highly prized in Mexico, where they are considered a traditional delicacy and command a high market value owing to their cultural, culinary, and nutritional significance (Molina-Vega et al. 2021; Herrera-Cardoso et al. 2025). This description not only highlights the presence of a potentially restricted-range species but also raises awareness for its conservation among land managers and researchers.

## Supplementary Material

XML Treatment for Aegiale

XML Treatment for Aegiale occidentalis

XML Treatment for Aegiale hesperiaris

## Additional information

### Use of AI

No use of AI was reported.

### Author ORCIDs

Sajan KC https://orcid.org/0000-0002-2749-0738

Keith R. Willmott https://orcid.org/0000-0002-9228-0219

Anisha Sapkota https://orcid.org/0000-0002-4351-6213

Vaughn M. Shirey https://orcid.org/0000-0002-3589-9699

### Data availability

The R code used to generate the species distribution model is publicly available on Figshare at https://figshare.com/s/1caed9410c2a7c392067 (https:/doi.org/10.6084/m9.figshare.31144999).

## References

[B1] Alducin-Martínez C, Ruiz Mondragón KY, Jiménez-Barrón O, Aguirre-Planter E, Gasca-Pineda J, Eguiarte LE, Medellin RA (2022) Uses, knowledge and extinction risk faced by agave species in Mexico. Plants 12(1): e124. 10.3390/plants12010124 PMC9824392 36616253

[B2] Bailowitz R, Brock J, Danforth D (2017) Annotated checklist of the butterflies (Lepidoptera) of Sonora, Mexico. Dugesiana 24(2): 125–147. 10.32870/dugesiana.v24i2.6639

[B3] Barnes W, McDunnough JH (1912) Contributions to the Natural History of the Lepidoptera of North America. Review Press, Decatur, Illinois, 43 pp. [6 pls.]

[B4] Blásquez P, Blásquez I (1865) Memoria Sobre el Maguey Mexicano (*Agave maximilianea*). Imprenta de Andrade, Mexico, 33 pp.

[B5] Bordelon C, Knudson E (2006) Illustrated checklist of Lepidoptera of the lower Rio Grande Valley (Vol. 1): Butterflies. TLS PUB. 9a. Texas Lepidoptera Survey, Houston, Texas, 60 pp.

[B6] Brock JP (2015) Field checklist of the butterflies of Sonora, Mexico [online]. http://www.mexicobirding.com/about/documents/ButterflyChecklist_Sonora_NonBooklet_2008.pdf

[B7] Chamberlain S, Oldoni D, Waller J (2022) rgbif: Interface to the Global Biodiversity Information Facility API. R package. 10.32614/cran.package.rgbif

[B8] Comstock JH (1918) The wings of insects: an exposition of the uniform terminology of the wing veins of insects and a discussion of the more general characteristics of the wings of the several orders of insects. Comstock Publishing Company, Ithaca, New York, 430 pp. 10.5962/bhl.title.54605

[B9] de la Maza RR (1987) Mariposas Mexicanas: Guía para su Colecta y Determinación. Fondo de Cultura Económica, Mexico City, Mexico, 302 pp.

[B10] Delgado-Lemus A, Torres I, Blancas J, Casas A (2014) Vulnerability and risk management of *Agave* species in the Tehuacán Valley, México. Journal of Ethnobiology and Ethnomedicine 10: 1–15. 10.1186/1746-4269-10-53 PMC4106216 24994025

[B11] Evans WH (1932) The Identification of Indian Butterflies (2^nd^ ed. rev. ed.). Bombay Natural History Society, Madras, 454 pp.

[B12] Evans WH (1949) A catalogue of the Hesperiidae from Europe, Asia and Australia in the British Museum (Nat. Mus.). Trustees of British Museum, London, xx + 502 pp. [53 pls.] 10.5962/bhl.title.105941

[B13] Evans WH (1955) A catalogue of the American Hesperiidae indicating the classification and nomenclature adopted in the British Museum (Natural History). Part IV. Hesperiinae and Megathyminae. Trustees of the British Museum (Natural History), London, 499 pp. [pls 54–88.]

[B14] Felder C, Felder R (1860) Lepidopterologische Fragmente. Wiener Entomologische Monatschrift 3: 263–273. 10.5281/zenodo.15984458

[B15] Folmer O, Black M, Hoeh W, Lutz R, Vrijenhoek R (1994) DNA primers for amplification of mitochondrial cytochrome c oxidase subunit I from diverse metazoan invertebrates. Molecular Marine Biology and Biotechnology 3(5): 294–299. 7881515

[B16] Freeman HA (1955) Four new species of *Megathymus* (Lepidoptera, Rhopalocera, Megathymidae). American Museum Novitates 1711: 1–10.

[B17] Freeman HA (1959) A revision of the genera of the Megathymidae, with descriptions of three new genera. Lepidopterists’ News 12(3/4): 81–92. [1958]

[B18] Freeman HA (1967) Speciation in the *Agathymus* (Megathymidae). Journal of Research on the Lepidoptera 5(4): 209–214. 10.5962/p.266929

[B19] Freeman HA (1969) Systematic review of the Megathymidae. Journal of the Lepidopterists’ Society 23(suppl. 1): 1–59.

[B20] Freeman HA, Hoffmann CC (1952) Two new species of *Megathymus* from Texas and Mexico (Lepidoptera, Rhopalocera, Megathymidae). American Museum Novitates 1593: 1–6.

[B21] Herrera-Cardoso ED, Tapia-Cervantes KA, Cepeda-Negrete J, Gutiérrez-Vargas S, León-Galván MF (2025) Isolation and identification of *Lactobacillus* species from gut microbiota of *Aegiale hesperiaris* (Lepidoptera: Hesperiidae) larvae. FEMS Microbiology Letters 372: fnaf015. 10.1093/femsle/fnaf015 39886864

[B22] Hijmans RJ, Phillips S, Leathwick J, Elith J (2017) dismo: Species distribution modeling. R package version 1.4: 1–1. 10.32614/cran.package.dismo

[B23] iNaturalist (2025) A Community for Naturalists · iNaturalist. https://www.inaturalist.org [Accessed 6 April 2025]

[B24] Jaimes-Rodríguez I, González-Hernández H, Llanderal-Cázares C, Rodríguez-Ortega A, Guzmán-Franco AW (2020) Traditional Mexican dish is associated with more than one skipper species (Lepidoptera, Hesperiidae, Megathiminae, Aegialini). Annals of the Entomological Society of America 113(3): 183–192. 10.1093/aesa/saz068

[B25] KC S, Sapkota A (2024) First record of the Mexican-M hairstreak *Parrhasius moctezuma* (Clench, 1971) (Lepidoptera: Lycaenidae) in Texas, USA, and possible sighting of the tequila giant skipper *Aegiale hesperiaris* (Walker, 1856) (Lepidoptera: Hesperiidae). Insecta Mundi 1084: 1–6.

[B26] Kirby WF (1877) A synonymic catalogue of diurnal Lepidoptera. Supplement, March, 1871–June, 1877. J. Van Voorst, London, pp. i–viii + 691–883. 10.5962/bhl.title.11413

[B27] Klots AB (1970) Lepidoptera. In: Tuxen SL (Ed.) Taxonomist’s Glossary of Genitalia in Insects (2^nd^ edn.). Munksgaard, Copenhagen, 115–130.

[B28] Molina-Vega A, Hernández-Domínguez EM, Villa-García M, Álvarez-Cervantes J (2021) *Comadia redtenbacheri* (Lepidoptera: Cossidae) and *Aegiale hesperiaris* (Lepidoptera: Hesperiidae), two important edible insects of *Agave salmiana* (Asparagales: Asparagaceae): a review. International Journal of Tropical Insect Science 41: 1977–1988. 10.1007/s42690-020-00396-1

[B29] Monteiro A, Pierce NE (2001) Phylogeny of *Bicyclus* (Lepidoptera: Nymphalidae) inferred from COI, COII, and EF-1α gene sequences. Molecular Phylogenetics and Evolution 18(2): 264–281. 10.1006/mpev.2000.0872 11161761

[B30] Piña-Domínguez IA, Ruiz-May E, Hernández-Rodríguez D, Zepeda RC, Melgar-Lalanne G (2022) Environmental effects of harvesting some Mexican wild edible insects: An overview. Frontiers in Sustainable Food Systems 6: e1021861. 10.3389/fsufs.2022.1021861

[B31] Posit Team (2024) RStudio: Integrated Development Environment for R. Posit Software, PBC. https://posit.co/

[B32] Ramos-Elorduy J (2006) Threatened edible insects in Hidalgo, Mexico and some measures to preserve them. Journal of Ethnobiology and Ethnomedicine 2: 1–10. 10.1186/1746-4269-2-51 PMC1716161 17144918

[B33] Ramos-Elorduy J, Moreno JM, Vázquez AI, Landero I, Oliva-Rivera H, Camacho VH (2011) Edible Lepidoptera in Mexico: geographic distribution, ethnicity, economic and nutritional importance for rural people. Journal of Ethnobiology and Ethnomedicine 7: 1–22. 10.1186/1746-4269-7-2 PMC3034662 21211040

[B34] Riley CV (1876) Notes on the yucca borer (*Megathymus yuccae* (Walk.)). Transactions of the Academy of Science of St. Louis 3: 323–344.

[B35] Roever K (1964) Bionomics of *Agathymus* (Megathymidae). Journal of Research on the Lepidoptera 3(2): 103–120. 10.5962/p.333477

[B36] Roever K (1975) In: Howe WH (Ed.) The Butterflies of North America. Doubleday and Co., Garden City, New York, 411–422.

[B37] Scott JA (1986) The Butterflies of North America: A Natural History and Field Guide. Stanford University Press, Stanford, California, 583 pp. 10.1515/9781503624450

[B38] Scudder SH (1875) Historical sketch of the generic names proposed for butterflies: a contribution to systematic nomenclature. Salem: Naturalist’s Agency; reprint from Proceedings of the American Academy of Arts and Sciences 10(2): 91–293. 10.5962/bhl.title.9688

[B39] Simon C, Frati F, Beckenbach A, Crespi B, Liu H, Flook P (1994) Evolution, weighting, and phylogenetic utility of mitochondrial gene sequences and a compilation of conserved polymerase chain reaction primers. Annals of the Entomological Society of America 87(6): 651–701. 10.1093/aesa/87.6.651

[B40] Vargas IF, Luis AM, Llorente JB, Warren AD (1996) Butterflies of the State of Jalisco, Mexico. Journal of the Lepidopterists’ Society 50: 97–138. 10.5281/zenodo.15935819

[B41] Walker F (1856) List of the Specimens of Lepidopterous Insects in the Collection of the British Museum. Part VII. British Museum (Natural History), London, 1509–1808.

[B42] Warren AD, Davis KJ, Stangeland EM, Pelham JP, Willmott KR, Grishin NV (2024) Illustrated Lists of American Butterflies (North and South America). https://www.butterfliesofamerica.com/index.html [Accessed 6 April 2025]

[B43] Willmott KR, Lamas G, Hall JPW (2017) Notes on the taxonomy of *Actinote intensa* Jordan (Lepidoptera: Nymphalidae: Heliconiinae) and the description of a new sibling species from eastern Ecuador. Tropical Lepidoptera Research 27(1): 6–15.

